# Exploratory Visual Analysis of Statistical Results from Microarray Experiments Comparing High and Low Grade Glioma

**Published:** 2007-04-01

**Authors:** David M. Reif, Mark A. Israel, Jason H. Moore

**Affiliations:** 1 Graduate Student, Computational Genetics Laboratory, Dartmouth-Hitchcock Medical Center, Lebanon, NH 03756; 2 Professor of Pediatrics and Genetics, Director, Norris-Cotton Cancer Center, Dart-mouth-Hitchcock Medical Center, Lebanon, NH 03756; 3 Frank Lane Research Scholar in Computational Genetics, Associate Professor of Genetics, 706 Rubin Building HB 7937; Dartmouth-Hitchcock Medical Center, One Medical Center Drive, Lebanon

**Keywords:** glioma, gene expression microarray, statistics, biological interpretation, visualization, annotation databases

## Abstract

The biological interpretation of gene expression microarray results is a daunting challenge. For complex diseases such as cancer, wherein the body of published research is extensive, the incorporation of expert knowledge provides a useful analytical framework. We have previously developed the Exploratory Visual Analysis (EVA) software for exploring data analysis results in the context of annotation information about each gene, as well as biologically relevant groups of genes. We present EVA as a flexible combination of statistics and biological annotation that provides a straightforward visual interface for the interpretation of microarray analyses of gene expression in the most commonly occuring class of brain tumors, glioma. We demonstrate the utility of EVA for the biological interpretation of statistical results by analyzing publicly available gene expression profiles of two important glial tumors. The results of a statistical comparison between 21 malignant, high-grade glioblastoma multiforme (GBM) tumors and 19 indolent, low-grade pilocytic astrocytomas were analyzed using EVA. By using EVA to examine the results of a relatively simple statistical analysis, we were able to identify tumor class-specific gene expression patterns having both statistical *and* biological significance. Our interactive analysis highlighted the potential importance of genes involved in cell cycle progression, proliferation, signaling, adhesion, migration, motility, and structure, as well as candidate gene loci on a region of Chromosome 7 that has been implicated in glioma. Because EVA does not require statistical or computational expertise and has the flexibility to accommodate any type of statistical analysis, we anticipate EVA will prove a useful addition to the repertoire of computational methods used for microarray data analysis. EVA is available at no charge to academic users and can be found at http://www.epistasis.org.

## Introduction

Modern experimental techniques such as microarray analysis of gene expression are improving our understanding of both the classification and biological basis of complex diseases. While there has been an explosion in the volume of raw data available for analysis, there is a widening gap between statistically compelling results and their biological interpretation. Research oftentimes becomes bogged down in an analytical maze of spreadsheets and arbitrary statistical significance thresholds. Tools are needed that can efficiently summarize huge amounts of information within a biological context. EVA’s novel combination of features supports such a comprehensive analysis. Combining flexibility, speed, and visualization of both statistical and annotative information into a single package, EVA fulfills a crucial role in comprehensive microarray analysis.

Here, we apply EVA to the study of glioma. These tumors arise in glial cells of the central nervous system and among many different functions, provide support, nourishment, and insulation to neurons in the brain (Odreman et al. 2005). Although rare, glioma account for approximately 45% of primary brain tumors (Legler et al. 1999). Astrocytoma, the most common subtype of glioma, arise in astrocytes. These tumors are classified into grades I–IV according to World Health Organization guidelines (Kleihues et al. 2002). Grade IV is highly-aggressive and also known as glioblastoma multiforme (GBM). Grade I is much less aggressive and commonly refered to as pilocytic astrocytoma. GBMs have a heterogeneous pathology and may develop from a lower-grade astrocytoma or appear to arise *de novo* (Odreman et al. 2005). Intermediate grades of glioma differ primarily in terms of their apparent proliferative rates and the aggressiveness with which they appear to infiltrate normal tissue. The treatment of varying grades of astrocytic glioma varies considerably, though the histologic basis on which these are differentiated is subtle and oftentimes confusing. The accurate diagnosis of glioma subclasses will become increasingly important as treatments become more specific and more effective. The elucidation of a molecular signature for such tumors, and especially GBM, should provide insights of both pathological and therapeutic significance and can be expected to enhance current diagnostic practice and facilitate the development of improved treatments.

## Methods

### Overview of the EVA software package

Visualization is the key design concept that allows EVA to rationally condense the vast amounts of information emerging from microarray based analyses of gene expression. In his work on visualizing quantitative information, Tufte states that “the most effective way to describe, explore, and summarize a set of numbers—even a very large set—is to look at pictures of those numbers” (Tufte 2001). With this philosophy in mind, EVA is controlled via a custom graphical user interface (GUI) that provides real-time updates to the global results display, while keeping all features of the software at the user’s fingertips. Through simple manipulation of both the density and type of information displayed, visual patterns of gene expression can be developed.

The user simply downloads the desktop client and requests a secure account, which provides a portal to the EVA web server via the custom GUI. This architecture provides ease of security, distributability, and expandability. Thus, once the user has downloaded the client, updates or expansions of the EVA modules are transparent from the user’s perspective. Upon opening the software, login grants the user access to interfaces for various administrative tasks, including creating, updating, deleting, or loading experiments and results. Individual users can access only those experiments for which they have permission, meaning that unless a group of collaborators has chosen to share a user account, a particular experiment is accessible only to the researcher who created it. Once a new experiment has been defined or an existing experiment selected, all of the results and links for that experiment are loaded for viewing on the user’s desktop.

EVA is designed to be flexible and support a wide range of research goals—allowing a truly exploratory analysis. The software can handle any kind of statistical result(s) for any number of experiments. Rather than being limited by those statistics implemented in the software, the user is free to use any statistic of choice or to define a custom statistic. When a file of gene or probe identifiers and associated statistical results is uploaded, the user specifies the parameters for each of the statistics in that file. Dialog boxes allow the user to choose amongst a list of pre-defined statistics (such as t-test p-value, fold change, etc.) or to define custom statistics representing that user’s particular analysis. EVA’s graphical results display can be organized into nested groupings for any combination of six biological categories: Gene Ontology (GO), Biopath, Domain, Map Location, Chromosome, and Phenotype (Harris et al. 2004; Kanehisa et al. 2004; Marchler-Bauer et al. 2003; McKusick-Nathans Institute for Genetic Medicine 2004; Pruitt and Maglott, 2001). The permutation testing procedure described in (Reif et al. 2005) is used to assess the statistical significance of certain biological groups that contain a higher proportion of differentially expressed genes relative to other groups. The permutation p-value represents the probability of obtaining the observed number of differentially expressed genes within the selected significance range in a given annotative category due to chance alone. As a complement to statistical analysis, EVA links to multiple annotation sources via Entrez Gene (Maglott et al. 2005). This aspect affords immediate evaluation of the biological relevance of candidate genes or groups of genes. To ensure that the user can replicate findings, EVA incorporates a printable command log feature into the GUI.

Speed enhances the interactivity of EVA’s flexible interface. Switching between annotative groupings, statistics, significance levels, and display modes occurs seamlessly, because all of the data and links are loaded into memory upon opening the software. While this design increases loading time, it allows immediate manipulation of the display once the initial loading is completed. Thus, performance is not limited by demand on the web server—negating the query lag time typical of other analysis packages.

A more complete description of the EVA interface and its capabilities is given in (Reif et al. 2005; Reif and Moore, 2006), and a comprehensive, illustrated help menu is included with the software. It is important to note that the EVA database is designed to store statistical results (e.g. p-values) and not raw microarray data. Since no patient identifying information is stored with the summary statistics, there are no confidentiality concerns with the database.

### Microarray data

The microarray dataset to which we applied EVA in this study is described fully in (Rickman et al. 2001) and is publicly available at the National Cancer Institute’s Gene Expression Data Portal (http://gedp.nci.nih.gov/dc/index.jsp). Forty primary glioma were obtained from surgery patients (with informed consent) at the University of Michigan Medical Center. Total RNA was isolated from these samples, which included 21 GBMs and 19 pilocytic astrocytomas. Expression was measured on HuGeneFL oligonucleotide chips from Affymetrix. Normalization and filtering were carried out as described in (Rickman et al. 2001), leaving approximately 4600 genes for statistical analysis.

### Application of EVA to glioma microarray results

**Figure 1.**
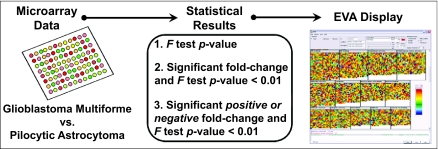
Genes whose expression levels discriminate between GBMs and pilocytic astrocytomas were identified using a combination of analysis of variance (ANOVA) and fold-change differences between tumor classes. These results were given to EVA as three separate statistics for each gene on the array: 1) the *F* test *p*-value, 2) the gene coded according to whether it had a significant fold-change and *p*-value < 0.01, and 3) the gene coded according to whether it had a significant *negative* fold-change and *p*-value < 0.01 or significant *positive* fold-change and *p*-value < 0.01.

Genes that discriminate GBMs from pilocytic astrocytomas were identified by the original authors using a combination of analysis of variance (ANOVA) and fold-change differences between tumor classes. One-way ANOVA models were fit with separate means for each tumor class. An *F* test was performed for each gene to test the hypothesis that the normalized mean expression of that gene is identical in both tumor classes. The resulting *p*-value represents the probability that the observed difference in expression between tumor classes is due to chance alone. As in (Rickman et al. 2001), fold-changes in measured expression levels of 1.5X or greater (p-value < 0.01) were considered significant. From these results, we defined three statistics for EVA. For each gene on the array, the three statistics loaded into EVA were: 1) the *F* test *p*-value, 2) the gene coded according to whether it had a significant fold-change and *p*-value < 0.01 or not, and 3) the gene coded according to whether it had a significant *negative* fold-change and *p*-value < 0.01, significant *positive* fold-change and *p*-value < 0.01, or neither. See Figure 1 for a summary of our application of EVA.

## Results

### EVA eliminates the traditional post-hoc assignment of biological relevancy

**Figure 2.**
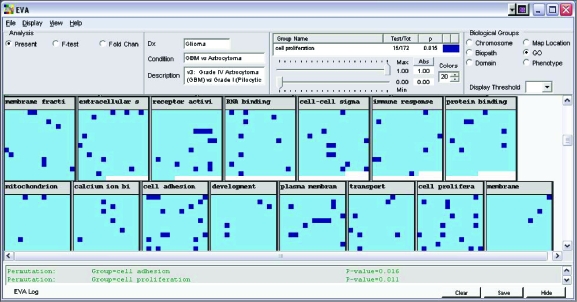
Each square represents the *p*-value for a particular gene, organized according to Gene Ontology. The “cell adhesion” and “cell proliferation” groups have a relatively high number of highlighted gene squares, meaning that they contain a number of genes that have a fold-change difference greater than 1.5 and *p*-value < 0.01. The results of permutation tests that statistically verify this visual impression are listed in the log.

By using EVA to examine the results of a relatively simple statistical analysis, we were able to identify tumor class-specific gene expression patterns having simultaneous statistical *and* biological significance (see Figure 2). In contrast, a typical microarray analysis demands a great deal of expertise in bioinformatics and requires the researcher to bridge the usual gap between statistical results and biological knowledge. EVA sheds light on findings that would have been missed by rigid statistical significance thresholds. The novel utility of EVA in bringing together a complementary set of visual, statistical, and annotative analysis tools is supported by the results described below.

### Biological patterns identified using EVA

Visualization of meaningful patterns in a sea of experimental results is EVA’s fundamental purpose. With the results organized according to Gene Ontology (GO), it is immediately apparent that the “cell adhesion” and “cell proliferation” groups have a relatively high number of highlighted gene squares (see Figure 2). Here, the EVA display is organized to highlight genes that have a fold-change difference greater than 1.5 and *p*-value < 0.01. Performing a permutation test on these categories statistically verifies this visual impression that the “cell adhesion” and “cell proliferation” groups are enriched with respect to the number of differentially expressed genes they contain. This conclusion also makes intuitive biological sense, since it is known that genes involved in cell adhesion and proliferation are differentially expressed in aggressive GBMs and low grade pilocytic astrocytomas (Muracciole et al. 2002).

The simple point-and-click interface for statistically testing the relative over-abundance of differentially expressed genes within certain biological groups is a useful mechanism for scanning an entire dataset in one sweep. Those groups having a greater proportion of differentially expressed genes than would be expected by chance are highlighted in the EVA log and can be written to an external file for further analysis. Using this strategy, interesting groups of genes involved in cellular migration, motility, and structure were identified. The GO groups “extracellular matrix,” “cytoskeleton organization and biogenesis,” and “metalloendopeptidase inhibitor activity,” as well as conserved protein Domain (from NCBI’s Conserved Domain Database) groups containing genes involved in cellular structure, were all significantly down-regulated. Aggressive tumors such as GBMs show altered expression of matrix metalloproteinases and other genes involved in regulation of cellular structure and motility (Odreman et al. 2005; Wick, Platten and Weller, 2001). Notably, other biologically plausible categories, such as the GO group “heterophilic cell adhesion” and the Domain group “Tubulin,” had permutation p-values just beyond the traditional 0.05 significance threshold and would have been missed by a purely statistical analysis. Employing EVA’s direct link that allows the annotation of individual genes (via Entrez Gene), we were able to identify biologically relevant candidate genes in these marginally significant groups. Many of the genes in these groups also had marginally significant individual statistical results. For example, disruption of microtubule dynamics by genes in the “Tubulin” group can affect apoptotic pathways (Yan et al. 2001).

EVA naturally incorporates expert knowledge into the statistical analysis via its biologically-organized display and links to gene-specific annotation. In an analysis of different histologic grades of brain tumors, it is reasonable to assume that differences in expression profiles of low- versus high-grade tumors might reflect alterations in cell cycle progression and cell signaling. Indeed, many differentially expressed genes appear in the related cell cycle GO groups “regulation of cell cycle,” “cytokinesis,” “mitosis,” and “oncogenesis” as well as the related cell signaling GO groups “JNK cascade,” “protein kinase activity,” and “GTPase activity.” Biopath groups “cell cycle” and “MAPK signaling” are also enriched. Additionally, Domain groups involving growth factor receptors contain many genes identified as significant. EVA’s permutation testing capabilities lend statistical significance to the observed high number of differentially expressed genes in these biological groups.

Further illustration of how EVA can tie together multiple lines of evidence in support of plausible biological conclusions or promising future research directions is the identification of regions on Chromosome 7 where candidate genes of interest might be located. Visual inspection and permutation testing identified chromosomal map region 7q21–7q32 as having a high frequency of differentially expressed genes. Chromosomal abnormalities on this chromosome have been implicated in glioma (Squire et al. 2001). This region includes the loci for Zyxin (a messenger in the signal transduction pathway that mediates adhesion-stimulated changes in gene expression) and HIPK2 (participates in the TGF-beta signaling pathway leading to JNK activation and apoptosis) (Hofmann et al. 2003; Li, Zhuang and Trueb, 2004). Corroborating evidence for the importance of genes in this region is provided by the membership of many genes within the GO and Biopath functional annotation groups mentioned above.

## Discussion

We have demonstrated that EVA provides a straightforward interface for incorporating biological knowledge into analysis of glioma microarray results. Using the same statistical results as (Rickman et al. 2001), EVA efficiently condensed volumes of data into a biologically-grounded graphical display—facilitating independent corroboration of the original authors’ conclusions, as well as identifying novel directions for glioma research. This novel combination of visualization, statistics, and biological knowledge in a fast, user-friendly package allowed for a truly comprehensive exploration of results.

EVA permits an analytical approach that is adaptable to the expertise or prior notions of the particular user and has the power to build cohesive conclusions supported by a collection of information sources. These capabilities are essential for confident analysis of complex microarray data, which has many methodological challenges, including the underestimation of actual changes in gene expression, temporal sensitivity, and the selection of appropriate probe sets (Yuen et al. 2002). A purely statistical analysis of gene expression data may give an incomplete portrayal of biological activity in the presence of such confounding issues. The multiple lines of evidence that EVA assembles can ameliorate some of these problems. EVA is ideal for efficiently exploring the biology behind statistical results, identifying promising avenues for further experimentation, and augmenting evidence for hypothesized biological models.

We anticipate EVA will be a useful addition to the repertoire of computational methods for microarray data analysis. Recent years have seen the development of many tools for gene expression analysis (Khatri et al. 2005), and while each fills a valuable analytical niche, EVA packages a set of exploratory tools that is appropriate for researchers with any level of statistical or computational expertise. Research into the continued refinement of EVA is ongoing. Future versions will link to additional gene expression databases, and EVA will be extended to accommodate experimental data from genetic and proteomic studies. The permutation algorithm for assessing the relative enrichment of annotative categories will be updated to account for issues of multiple testing and correlation, following approaches similar to (Barry et al. 2005). EVA is available at no charge to academic users. More information about the continued development of EVA can be found at http://www.epistasis.org.

## Abbreviations:

EVA: Exploratory Visual Analysis;
GBM: glioblastoma multiforme;
GUI: graphical user interface;
GO: Gene Ontology;
ANOVA: analysis of variance.
